# Alteration of protein prenylation promotes spermatogonial differentiation and exhausts spermatogonial stem cells in newborn mice

**DOI:** 10.1038/srep28917

**Published:** 2016-07-04

**Authors:** Fan Diao, Chen Jiang, Xiu-Xing Wang, Rui-Lou Zhu, Qiang Wang, Bing Yao, Chao-Jun Li

**Affiliations:** 1 MOE Key Laboratory of Model Animals for Disease Study, Model Animal Research Center and the Medical School of Nanjing University, National Resource Center for Mutant Mice, Nanjing 210061, China; 2 Center of Reproductive Medicine, Nanjing Jinling Hospital, the Medical School of Nanjing University, Nanjing 210002, China

## Abstract

Spermatogenesis in adulthood depends on the successful neonatal establishment of the spermatogonial stem cell (SSC) pool and gradual differentiation during puberty. The stage-dependent changes in protein prenylation in the seminiferous epithelium might be important during the first round of spermatogenesis before sexual maturation, but the mechanisms are unclear. We have previous found that altered prenylation in Sertoli cells induced spermatogonial apoptosis in the neonatal testis, resulting in adult infertility. Now we further explored the role of protein prenylation in germ cells, using a conditional deletion of geranylgeranyl diphosphate synthase (Ggpps) in embryonic stage and postmeiotic stage respectively. We observed infertility of *Ggpps*^*−/−*^ Ddx4-Cre mice that displayed a Sertoli-cell-only syndrome phenotype, which resulted from abnormal spermatogonial differentiation and SSC depletion during the prepubertal stage. Analysis of morphological characteristics and cell-specific markers revealed that spermatogonial differentiation was enhanced from as early as the 7^th^ postnatal day in the first round of spermatogenesis. Studies of the molecular mechanisms indicated that *Ggpps* deletion enhanced Rheb farnesylation, which subsequently activated mTORC1 and facilitated spermatogonial differentiation. In conclusion, the prenylation balance in germ cells is crucial for spermatogonial differentiation fate decision during the prepubertal stage, and the disruption of this process results in primary infertility.

Continuous spermatogenesis in male mammals is maintained by a supply of differentiating cells from self-renewing SSC pool throughout the reproductive age^1^, which is established within a few days after birth in mice. These pivotal events of spermatogenesis, including establishment of the “SSC pool”, the differentiation of spermatogonia and the initiation of meiosis during prepubertal stage, are precisely co-regulated by germ cells and the SSC niche^2^. Any impairment of these processes because of particular mutant and genetically modification would result in primary infertility. For instance, mutations in Gdnf (or Ret and Gfra1) induced progressive germ cell loss due to a depletion of stem cell reserves, whereas GDNF overexpression leads to the accumulation of undifferentiated spermatogonia^3^,^4^,^5^. Loss of SCF or c-kit function disrupts spermatogonia differentiation and promotes A_undiff_ spermatogonia accumulation and apoptosis^6^,^7^,^8^,^9^. These defects arise during the first round of spermatogenesis before puberty, could impair testicular development and cause primary sterility in adult males.

In rat seminiferous epithelium, isoprenoid modification and prenylated protein levels correlate with different spermatogenesis events. Protein prenyltransferase (PFT and PGGT-I) activities increased during the differentiation of spermatogonia in prepubertal ages, peaked at postnatal days 9 and 23, and then decreased after sexual maturity. Meanwhile, total protein prenylation and the ratio of geranylgeranylated to farnesylated protein decreased after postnatal day 9, and continued to decrease as age increased^10^. Protein prenylation, including farnesylation and geranylgeranylation, is an important protein modification that can covalently attach either a farnesyl diphosphate (FPP) or geranylgeranyl diphosphate (GGPP) to conserved cysteine residues at or near the C-terminus of particular proteins^11^. Both FPP and GGPP are important intermediates in the mevalonate pathway, and the branch point enzyme Ggpps can synthesize GGPP by adding an isoprenoid to FPP^11^. In studies focused on the side effects of statins in children with dyslipidemias, the inhibition of the mevalonate pathway with the HMG-CoA reductase inhibitor rosuvastatin delayed pubertal male rat reproductive development and structural damage to the epididymis and testis^12^. Moreover, our study of male infertility patients who had been infected with the mumps virus before puberty, altered prenylation levels caused by Ggpps deficiency in Sertoli cells induced excessive cytokine and chemokine synthesis and secretion, which resulted in spermatogonia apoptosis and subsequent infertility in adult mice^13^. These findings suggest that protein prenylation in the seminiferous epithelium is crucial in early stage of spermatogenesis before sexual maturity. However, the particular role and regulated mechanism of protein prenylation in germ cells during spermatogenesis still remains unclear.

When responding to GDNF, SCF and retinoic acid (RA) signals, the PI3K/AKT/mTOR signaling network is essential for maintaining stem cell homeostasis^14^,^15^,^16^. Aberrant Akt/mTOR signaling activation could trigger built-in cellular fail-safe mechanisms by altering downstream gene translation to induce apoptosis and cause stem cell depletion^17^,^18^. It has recently been reported that mTORC1 signaling plays a key instructive role in spermatogonial progenitor cell (SPC) maintenance and differentiation^16^. Evidence suggests that mTORC1 activity requires the Ras-like small GTPase Rheb^19^,^20^,^21^. Indispensable, Rheb is targeted to endomembranes via farnesylation in its C-terminal CAAX motif 22 and its activation is opposed by the tuberous sclerosis heterodimer complex (TSC1/TSC2), which directly promotes the conversion of Rheb-GTP to Rheb-GDP^23^,^24^,^25^. Our previous study demonstrated that *Ggpps* deletion in cardiomyocytes disrupted the balance between protein farnesylation and geranylgeranylation^26^. Elevated Rheb farnesylation subsequently activated mTORC1 signaling and induced cardiomyocyte hypertrophy, cardiac fibrosis and excessive apoptosis, eventually led to severe heart failure^26^.

In light of these findings, we hypothesized that prenylation of the small GTPase might play a particular role in spermatogenesis within germ cells. In this study, Ddx4-Cre (Vasa-Cre) and Prm1-Cre were crossed with Ggpps-floxed mice respectively, to generate Ggpps germ cell-specific knockout mice at different stages of spermatogenesis. Phenotypic analysis which focused on the first round of spermatogenesis since neonatal stages of conditional knockout mouse revealed that altered protein prenylation impaired spermatogonial differentiation and induced SSC depletion. Mechanistic studies further proved that protein farnesylation levels contributed to spermatogonial differentiation mediated by the Rheb-activated mTORC1 pathway.

## Results

### *Ggpps* deletion in germ cells results in complete loss of germ cells and male infertility

**Figure 1:**
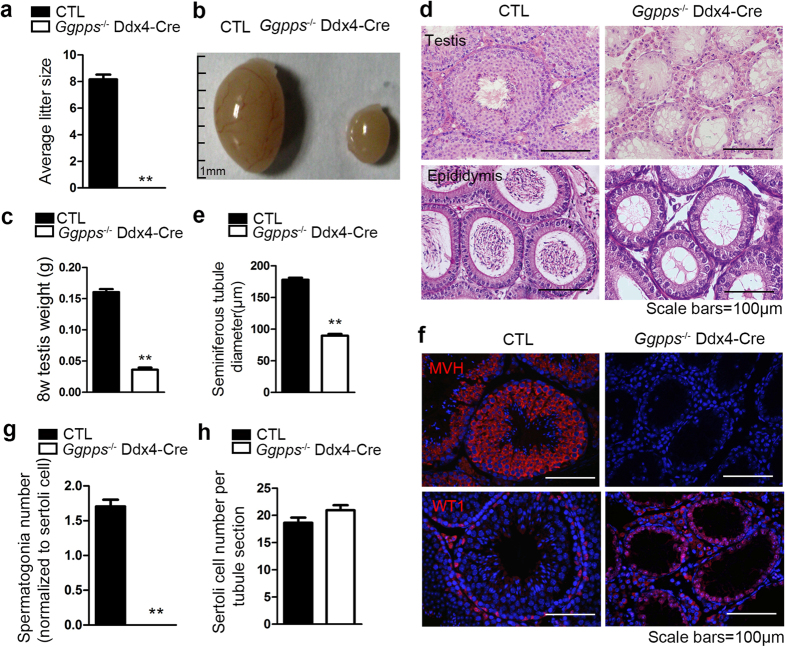
*Ggpps* deletion in germ cells leads to complete infertility and germ cell loss in adult male mice (8 weeks postnatal). (**a**) Mating test litter size shows that *Ggpps*^*−/−*^ Ddx4-Cre adult mice have no offspring. n = 6, **p = 5.82E-10. (**b**) Difference in the appearance between CTL and *Ggpps*^*−/−*^ Ddx4-Cre testes. Scale bar = 1 mm (**c**) Weight differences in testes of CTL and *Ggpps*^*−/−*^ Ddx4-Cre testes. n = 6, **p = 3.03E-10. (**d**) H&E staining of testes transections and the epididymal lumen shows atrophied seminiferous tubules and an absence of mature spermatozoa in *Ggpps*^*−/−*^ Ddx4-Cre mice. Scale bar = 100 μm. (**e**) Mean seminiferous tubule diameter measurements of CTL and *Ggpps*^*−/−*^ Ddx4-Cre mice. n = 5, **p = 1.97E-10. (**f**) MVH and Wt1 immunofluorescence in testis sections of CTL and *Ggpps*^*−/−*^ Ddx4-Cre mice. Scale bar = 100 μm (**g**) Quantification and statistical analysis of spermatogonia number normalized to sertoli cell show severe germ cell loss in *Ggpps*^*−/−*^ Ddx4-Cre mice. n = 5, **p = 1.71E-14. (**h**) Statistical analysis of Sertoli cell number per tubule section indicates that *Ggpps*^*−/−*^ germ cell knockout does not affect Sertoli cells. n = 5.

We previously found that *Ggpps* deletion in Sertoli cells altered GGPP and FPP levels, which caused germ cell loss through abnormal cytokine and chemokine release^13^. And the detection of the expression pattern of *Ggpps* in seminiferous tubules by immunofluorescence showing its high expression in sertoli cells, spermatogonia and primary spermatocytes (arrows) (Fig. S1a). To assess the possible functions of GGPP and FPP in germ cells, we constructed germ cell-conditional *Ggpps* knockout mice by crossing *Ggpps*-floxed mice with Ddx4-Cre mice. Ddx4-Cre induces recombination in germ cells between E15 to E18, and thus, deletes *Ggpps* in all stages of spermatogenetic cells^27^. And the *Ggpps* knockout efficiency was verified on mRNA level and protein level in isolated germ cells (Fig. S1b,c). We also confirmed that *Ggpps* deletion in germ cells results in GGPP deficiency and FPP accumulation by HPLC–MS/MS analysis (Fig. S1d,e). To determine whether Ggpps deficiency in germ cells had any impact on male fertility, 8-week-old male knockout mice and their control littermates were mated with wild-type (C57BL/6) females for period of 4 months. All control males produced a normal amount of offspring over this period, but *Ggpps*^*−/−*^ Ddx4-Cre males were completely infertile (Fig. 1a). Examination of adult male testes revealed a significant decrease in testis size and weight in *Ggpps*^*−/−*^ Ddx4-Cre mice compared to controls (Fig. 1b,c). Histological analysis revealed severe germ cell loss in 8-week-old *Ggpps*^*−/−*^ Ddx4-Cre testes (Fig. 1d). In 8-week-old control mice, the spermatogenetic cells which in progressive stages, orderly arranged concentrically from the basement to the center of the seminiferous tubules, and numerous mature spermatozoa in the epididymal lumen (Fig. 1d). In contrast, *Ggpps*^*−/−*^ Ddx4-Cre mice displayed atrophied seminiferous tubules with a mean diameter reduced by 50% and only a single layer of cells around the basement membrane (Fig. 1d,e). In addition, we did not observe any mature spermatozoa in the epididymides of *Ggpps*^*−/−*^ mice (Fig. 1d). Furthermore, immunofluorescence staining with the germ cell marker MVH and Sertoli cell marker WT1 indicated that *Ggpps*^*−/−*^ Ddx4-Cre mice had no germ cells in seminiferous tubule and displayed a Sertoli cell only syndrome phenotype (Fig. 1f–h). Together, these data indicated that *Ggpps* deletion in germ cells resulted in germ cell loss and seminiferous tubule degeneration, which led to male infertility.

### The effect of Ggpps is restricted in the early stages of spermatogenesis before differentiation

**Figure 2:**
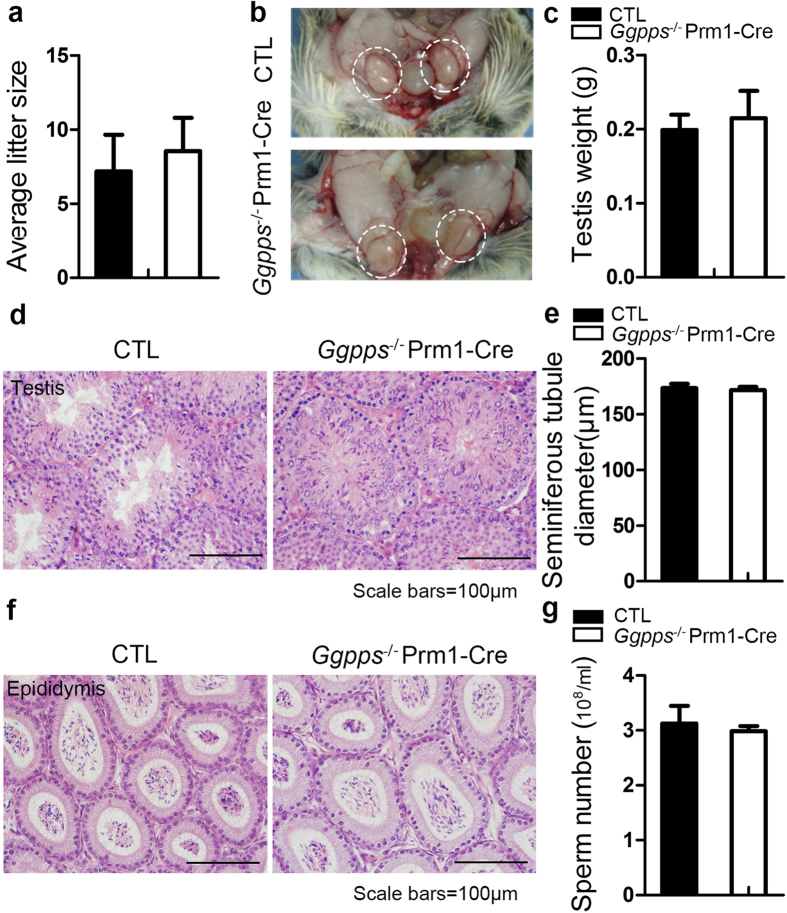
Adult male mice with *Ggpps* deletion in haploid spermatids show normal reproductive ability compared to control mice (8 weeks postnatal) (**a**) Mating test litter size showing *Ggpps*^*−/−*^Prm1-Cre mice have a normal number of offspring. n = 6 (**b**) Testis appearance and (**c**) weight are similar in CTL and *Ggpps*^*−/−*^ Prm1-Cre mice. n = 6. (**d**) H&E staining of testis transections in CTL and *Ggpps*^*−/−*^ Prm1-Cre mice both show regular seminiferous tubules with spermatogenic cells at all stages. Scale bars = 100 μm (**e**) Mean seminiferous tubule diameter measurements. n = 5. (**f**) H&E staining of epididymal lumen of CTL and Ggpps^*−/−*^ Prm1-Cre testes. (**g**) Sperm counts displaying no significant differences between CTL and *Ggpps*^*−/−*^ Prm1-Cre mice. n = 5.

We noticed that Ggpps is highly expressed in spermatogonia located atop the basement membrane of seminiferous tubules, but is weakly expressed or not expressed in secondary spermatocytes (asterisk) (Fig. S1a). In subsequent experiments, we crossed Prm1-Cre mice with *Ggpps*-floxed mice to delete *Ggpps* in the spermatogenic cells at the haploid spermatid stage after differentiation. And the *Ggpps* knockout efficiency was verified on mRNA level and protein level in isolated germ cells (Fig. S1b,c). *Ggpps*^*−/−*^ Prm1-Cre mice showed a phenotype similar to control mice. Fertility assays indicated there were no differences in litter size between knockout mice and their control littermates (Fig. 2a). Testis weight and morphology were also similar (Fig. 2b,c). H&E staining did not show any differences in cellular morphology between knockout mice and their control littermates. Spermatogenetic cells were organized in a strict order of maturation towards the lumen (Fig. 2d), and the seminiferous tubules were normal in diameter (Fig. 2e). The morphology of the epididymides and the sperm count of adult knockout mice were also normal (Fig. 2f,g). The unaffected fertility of *Ggpps*^*−/−*^ Prm1-Cre mice suggests that *Ggpps* regulated protein prenylation only affects spermatogenesis before differentiation stage.

### *Ggpps* deletion in germ cells results in developmental defects as early as the first round of gametogenesis

**Figure 3:**
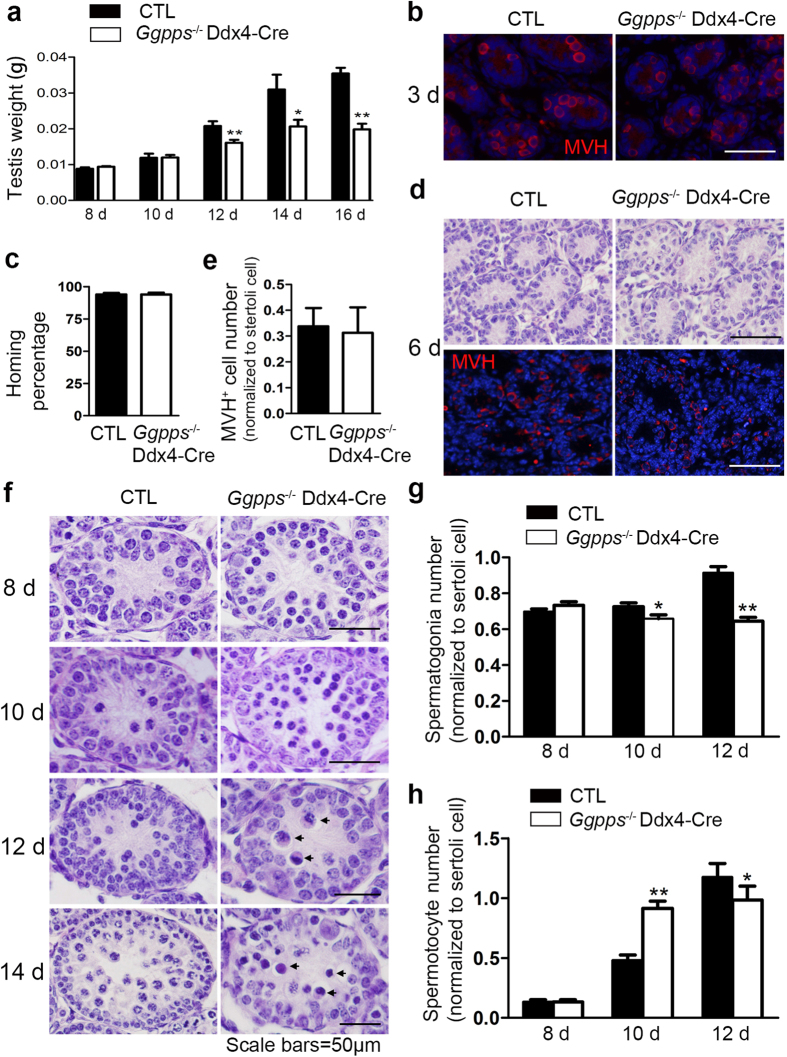
Infertility of *Ggpps*^*−/−*^ Ddx4-Cre male mice is a consequence of germ cell depletion within the first round spermatogenesis. (**a**) Testis weight of CTL and *Ggpps*^*−/−*^ Ddx4-Cre mice between 8 d and 16 d postnatal. n = 5, 12 d **p = 5.90E-04, 14 d *p = 3.44E-02, 16 d **p = 6.41E-03. (**b**) MVH immunofluorescence of testes transections of 3 d CTL and *Ggpps*^*−/−*^ Ddx4-Cre mice. Scale bars = 50 μm. (**c**) Homing percentage of spermatogonia in 3 d CTL and *Ggpps*^*−/−*^ Ddx4-Cre mice. n = 3. (**d**) H&E staining and MVH immunofluorescence in 6 d CTL and *Ggpps*^*−/−*^ Ddx4-Cre mice. Scale bar = 50 μm. (**e**) MVH^+^ cell number of 6 d CTL and *Ggpps*^*−/−*^ Ddx4-Cre mice normalized to sertoli cell. n = 5. (**f**) H&E staining of testes transections showing typical morphological differences between CTL and *Ggpps*^*−/−*^ Ddx4-Cre mice between 8 d and 14 d postnatal. Arrows indicate shrinking and hyperchromatic spermatocytes. Scale bar = 50 μm. (**g**) Identification and statistical analyses of spermatogonia number normalized to sertoli cell between 8 d and 12 d postnatal. n = 5, 10 d *p = 3.98E-02, 12 d **p = 4.30E-06. (**h**) Identification and statistical analyses of spermatocytes number normalized to sertoli cell between 8 d and 12 d postnatal. n = 5, 10 d **p = 1.17E-07, 12 d *p = 3.21E-02.

To determine the initial time point of the spermatogenetic defect caused by *Ggpps* deletion, we examined the testicular weight of newborn mice and found a significant decrease occurred as early as postnatal day 12 in *Ggpps*^*−/−*^ Ddx4-Cre mice (Fig. 3a). There are several critical stages of spermatogenesis that occur before postnatal day 12. During the homing process in postnatal days 0–3, gonocytes migrate to the basal membrane, transform into SSCs and become tightly enfolded by one or more somatic Sertoli cells to form a niche^28^,^29^. Spermatogonia proliferate through mitosis during postnatal days 3–6, followed by the first round of differentiation from postnatal day 7^2^. We found that over 95% of spermatogonia which marked by MVH immunofluorescence localized to the basement membrane of seminiferous tubules in control and *Ggpps*^*−/−*^ Ddx4-Cre mice at postnatal day 3, which indicated that the homing ability of SSC was unaffected by loss of *Ggpps* (Fig. 3b,c). H&E staining and MVH immunofluorescence indicated that there was no significant difference in cell number of spermatogonia between control and *Ggpps*^*−/−*^ Ddx4-Cre mice at postnatal day 6 (Fig. 3d,e). The identification and quantitative analysis of spermatogonia and spermatocytes in each tubule section indicated that spermatogonia decreased from postnatal day 10 (Fig. 3f,g) and the spermatocyte first increased from postnatal days 10 and then subsequently decreased at postnatal day 12 in *Ggpps*^*−/−*^ Ddx4-Cre mice (Fig. 3h). Moreover, *Ggpps*^*−/−*^ Ddx4-Cre mice showed increased shrinking and hyperchromatic spermatocytes (Fig. 3f, indicated by arrows), which might indicate apoptosis and be responsible for the decrease in spermatocyte number in *Ggpps*^*−/−*^ Ddx4-Cre mice after postnatal day 12 (Fig. S2a,b). These data indicate that Ggpps deficiency in germ cells results in disturbance of the transition from spermatogonia to spermatocytes and induces a mass of apoptosis during the first round spermatogenesis of newborn mice.

### Germ cell deletion of *Ggpps* induces excessive differentiation and exhaustion of SSC

**Figure 4:**
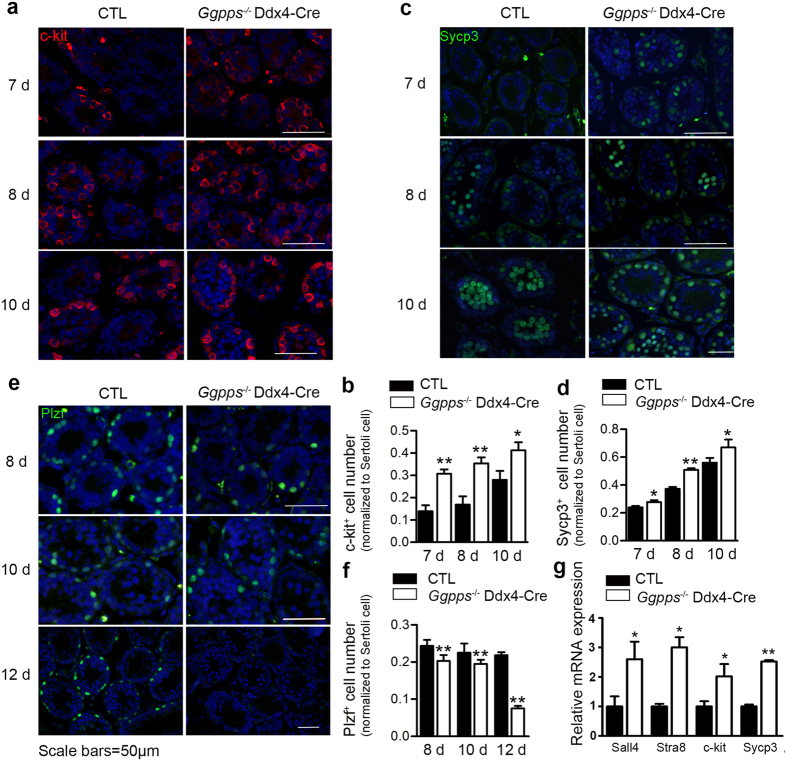
*Ggpps*^*−/−*^ Ddx4-Cre mice display enhanced spermatogonial differentiation from postnatal day 7, followed by excessive meiosis entry, and precocious exhaustion of the SSC pool (**a,c,e**) immunofluorescence of the following marker genes: c-kit, Sycp3 and Plzf in tubule sections between postnatal 7 d and 12 d. Scale bars = 50 μm. (**b**) Statistical analysis of c-kit-positive spermatogonia normalized to sertoli cell between 7 d to 10 d shows the enhanced differentiation of *Ggpps*^*−/−*^ Ddx4-Cre mice. n = 5, 7 d **p = 5.98E-05, 8 d **p = 8.11E-04, 10 d *P = 2.79E-02. (**d**) Statistical analysis of Sycp3-positive spermatogenetic cells normalized to sertoli cell between 7 d and 10 d shows the precocious and excessive meiotic cells in *Ggpps*^*−/−*^Ddx4-Cre mice. n = 5, 7 d *p = 3.76E-02, 8 d **p = 4.815E-07, 10 d *p = 4.50E-02. (**f**)Statistical analysis of Plzf-positive spermatogonia normalized to sertoli cell shows a decrease in the number of undifferentiated spermatogonia in *Ggpps*^*−/−*^Ddx4-Cre mice. n = 5, 8 d **p = 2.50E-03, 10 d **p = 1.43E-03, 12 d **p = 2.71E-05 (**g**) Relative mRNA expression spermatogonia differentiation and meiosis-specific marker genes Sall4, Stra8, c-kit, and Sycp3 is increased in spermatogenetic cells from *Ggpps*^*−/−*^Ddx4-Cre mice. Sall4 *p = 4.36E-02, Stra8 *p = 3.88E-02, c-kit *p = 5.12E-03, Sycp3 **p = 6.54E-04.

The decrease in spermatogonia and increase in spermatocytes after postnatal day 10 in the *Ggpps*^*−/−*^ Ddx4-Cre mice provides clues that loss of *Ggpps* in germ cells enhanced spermatogonial differentiation. To confirm this hypothesis, we performed immunofluorescence using the differentiation marker c-kit and observed that the number of c-kit^+^ spermatogonia increased in *Ggpps*^*−/−*^ Ddx4-Cre tubules beginning at postnatal day 7 (Fig. 4a,b). The immunofluorescence of SYCP3, a synaptonemal complex protein that is primarily expressed in the lateral portion of the synaptonemal complex^30^,^31^ also showed that the number of meiotic cells in each tubule section significantly increased as early as postnatal day 7 in *Ggpps*^*−/−*^ Ddx4-Cre mice (Fig. 4c,d). Furthermore, almost all germ cells around the basement membrane of the tubules were SYCP3^+^ at postnatal day 10 (Fig. 4c). Meanwhile, we performed immunofluorescence of Plzf, which is a marker of undifferentiated spermatogonia (including SSCs) and found that the number of Plzf^+^ cells decreased starting at postnatal day 8 and was almost disappeared at postnatal day 12 in *Ggpps*^*−/−*^ Ddx4-Cre mice (Fig. 4e,f). To further examine and compare the extent of differentiation, we isolated spermatogenic cells from the testes of *Ggpps*^*−/−*^ Ddx4-Cre and wild-type mice at postnatal day 7 and detected mRNA expression of specific differentiation and meiosis related genes. We found a significant up-regulation of Sall4, Stra8, c-kit, and Sycp3, supporting the enhanced differentiation in *Ggpps*^*−/−*^ Ddx4-Cre mice (Fig. 4g).These data above suggested that *Ggpps* deletion results in excessive spermatogonia differentiation and leads to the depletion of SSC.

### Germ cell deletion of *Ggpps* enhances mTORC1 signaling by increasing Rheb farnesylation

**Figure 5:**
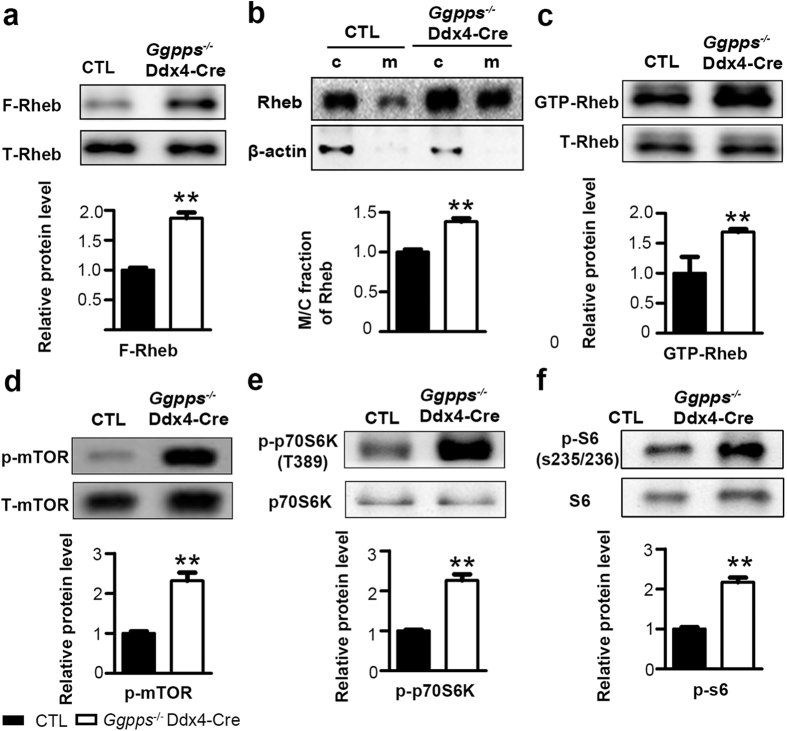
Germ cell deletion of *Ggpps* enhances Rheb farnesylation and induces spermatogonia differentiation via the mTORC1 pathway. (**a**) Rheb farnesylation increased in *Ggpps*^*−/−*^ Ddx4-Cre spermatogenic cells. **p = 1.01E-03 (**b**) Rheb membrane association increased in *Ggpps*^*−/−*^Ddx4-Cre spermatogenic cells. **p = 1.88E-03 (**c**) Activated GTP-bound Rheb increased in *Ggpps*^*−/−*^ Ddx4-Cre spermatogenic cells. **p = 6.73E-03 (**d–f**) Western blot analysis was performed for p-mTOR (Ser2448), mTOR, p-p70S6K (Thr389), p70S6K, p-S6 (Ser235/236) and S6, showing hyper activation of the mTORC1 pathway. P-mTOR **p = 3.19E-03, p-p70S6K **p = 6.34E-05, p-p70 **p = 8.06E-04.The gels were run under the same experimental conditions. The full-length blots are presented in Supplementary Figs 3–8. Relative protein levels determined by density analysis and normalized by corresponding total protein control.

It has previously been reported that the mTORC1 pathway is critical for regulating SSC self-renewal and differentiation^16^,^18^. Additionally, farnesylation is essential for Rheb membrane localization and activation, which is required in activation of mTORC1 signaling pathway^26^,^32^,^33^,^34^. We have already confirmed the excessive differentiation of *Ggpps*^*−/−*^ Ddx4-Cre testicular tissue at postnatal day 7 by immunofluorescence and mRNA analysis. We next isolated spermatogenetic cells from the testes at postnatal day 7 to assess Rheb/mTORC1 pathway activity at the protein level. As expected, Rheb farnesylation increased after *Ggpps* deletion in germ cells (Fig. 5a). Enhanced farnesylation facilitated the membrane association of Rheb (Fig. 5b), which resulted in an increased proportion of active GTP-bound Rheb (Fig. 5c). As a result, mTOR was phosphorylated and activated (Fig. 5d), and phosphorylation of its downstream targets p70S6K and S6 was up-regulated in spermatogenetic cells isolated at postnatal day 7 (Fig. 5e,f). These results suggest that enhanced Rheb farnesylation in germ cells leads to hyper activation of the mTORC1 pathway in *Ggpps*^*−/−*^ Ddx4-Cre mice.

### Blocking mTORC1 signaling rescues SSC exhaustion induced by *Ggpps* deletion

**Figure 6:**
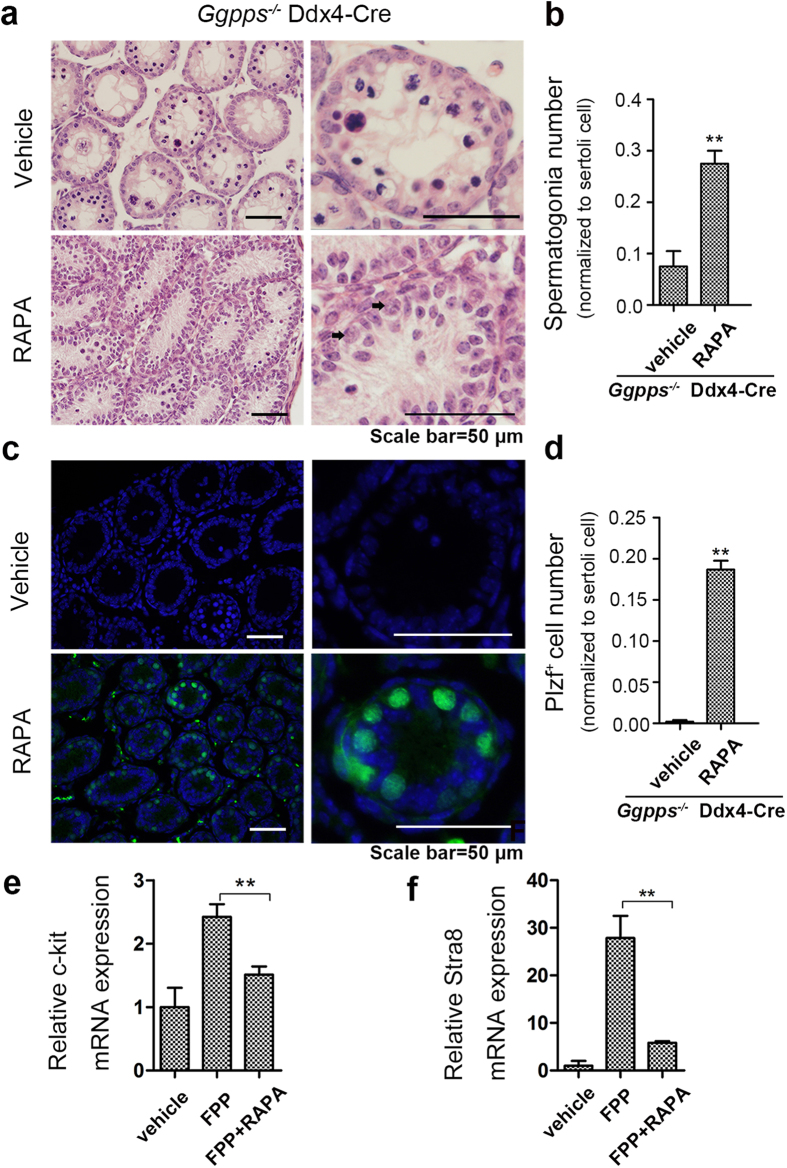
mTORC1 pathway inhibition suppresses Ggpps deficiency-induced SSC exhaustion. (**a**) H&E staining of testis transections showing typical morphological differences of vehicle and drug-treated groups at 14 d postnatal. Arrows indicate spermatogonia. Scale bar = 50 μm (**b**) Statistical analysis of spermatogonia number in 14 d postnatal mice in vehicle and drug-treated groups, which were normalized to sertoli cell. n = 3, **p = 2.01E-04 (**c**) Plzf immunofluorescence of testis transections from 14 d postnatal mice in vehicle and drug-treated groups. Scale bars = 50 μm. (**d**) Statistical analysis of Plzf-positive spermatogonia number in 14 d postnatal mice in vehicle and drug-treated groups, which were normalized to sertoli cell. n = 3, **p = 1.41E-04 (**e,f**) Relative mRNA expression of the marker genes c-kit and Stra8 after *in vitro* treatment of isolated Thy1^+^ spermatogenetic cells with FPP and rapamycin alone or in combination. c-kit **p = 2.35E-04, Stra8 **p = 4.72E-04.

To confirm that mTORC1 activation induced by *Ggpps* deletion is responsible for the excessive spermatogonial differentiation and SSC exhaustion, we injected the mTORC1 inhibitor rapamycin to check the resumption of spermatogenesis of Ddx4-Cre knockout mice. We treated the *Ggpps*^*−/−*^ Ddx4-Cre mice with intraperitoneal injection of rapamycin or vehicle every 24-hour from postnatal day 3 through day 14. Then we found the number of spermatogonia (arrows) increased in rapamycin-injected *Ggpps*^*−/−*^ Ddx4-Cre mice compared to vehicle treated knockout mice (Fig. 6a,b). In addition, the number of Plzf^+^ spermatogonia located at the basement membrane of seminiferous tubules also increased, which confirmed that rapamycin rescued undifferentiated spermatogonia (including SSCs) in *Ggpps*^*−/−*^ Ddx4-Cre mice (Fig. 6c,d). Moreover, rapamycin inhibited FPP-induced c-kit and Star8 expression *in-vitro* (Fig. 6e,f). In conclusion, blocking the mTORC1 pathway inhibited the expression of differentiation related genes and rescued SSC exhaustion caused by increased Rheb farnesylation in germ cells.

## Discussion

The spermatogonial differentiation involves the orchestration of a serial of interconnected networks and also requires the physical support of somatic cells and signal-based stimulation, such as growth factors, chemotactic factors, and steroid hormones in the niche microenvironment^35^,^36^. However, the intrinsic molecular mechanism of germ cells that determines the differentiation of spermatogonia still remains to be further explored.

In this report, we showed that the protein prenylation balance takes part in spermatogonial differentiation of newborn mice. *Ggpps* deletion in germ cells causes FPP accumulation, enhances Rheb farnesylation and activates the mTORC1 pathway, which accelerates differentiation. The excessive differentiation of spermatogonia induces high levels of apoptosis, which causes the exhaustion of SSC pool and germ cell loss at the early stage of the first round of spermatogenesis. However, *Ggpps* deletion in haploid spermatids using Prm1-Cre did not affect the fertility of male mice, which suggested that Ggpps-regulated protein prenylation balance functions only during the early stages of spermatogenesis. During testicular development of newborn mice, GGPP and FPP significantly increase in germ cells at postnatal day 8 compared to day 6 (Fig. S1d,e), which would promote various biological processes by activating small GTPase and their downstream pathways^37^,^38^,^39^,^40^. Our finding that GGPP deficiency and increased FPP in *Ggpps*-Ddx4-cre knockout mice would result in SSC exhaustion suggested that the balance between protein geranylgeranylation and farnesylation is critical for spermatogonial differentiation.

The germ cell and the niche factors precisely co-regulate the differentiated fate decision of SSC. Retinoic acid (RA) stimulates the translation of Kit mRNAs by binding to RARs and activating PI3K/AKT/mTOR signaling^41^,^42^. SCF/c-kit responds to RA stimulation and promotes cell differentiation by up-regulating Cyclin D3 through the PI3K/AKT/mTOR pathway in spermatogonia^41^,^43^. Moreover, Stra8 responds to RA through the PI3K/AKT/mTOR pathway and functions in DNA replication of germ cells, which facilitates the differentiation of spermatogonia^44^,^45^. In summary, PI3K/AKT/mTOR signaling regulates spermatogonial differentiation, which are pivotal for SSC fate determination. According to our data, the increase in Rheb farnesylation activated the mTORC1 pathway and promoted spermatogonial differentiation by inducing c-kit and Stra8 expression. These findings suggest that Rheb farnesylation acts as an “ON-OFF switch” that regulates mTORC1 activation in SSC fate regulation for the stimulation of differentiation during spermatogenesis.

Statins have been widely used to treat dyslipidemia, as they prevent the conversion of HMG-CoA into mevalonate, which decreases endogenous cholesterol formation and inhibits the synthesis of other important compounds^46^. However, related clinical analyses have shown that statin therapy might induce an overt primary hypogonadism in adult males, causing decreased testosterone levels associated with down regulation of penile RhoA/Rho-kinase (ROCK) signaling^47^,^48^. Some studies have found that pubertal male rats exposed to rosuvastatin since pre-puberty showed delayed reproductive development^12^. These side effects of statins are attributable to alterations in cholesterol and lipid metabolism in both children and adults. According to our work, it is also possible that some intermediates of cholesterol synthesis, such as lanosterol or the isoprenoids GGPP and FPP, which are involved in spermatogonia differentiation and meiosis initiation, are influenced by the upstream inhibition of the mevalonate pathway. It is essential to study the mechanism of Ggpps-regulated prenylation within the mevalonate pathway during spermatogenesis to determine effective drug targets and to avoid the side effects of dyslipidemia treatment and to treat primary male sterility.

The present study demonstrated that protein farnesylation is an important and comprehensive cell-intrinsic regulator, and it promotes transcription and expression of differentiation-related genes through the mTORC1 signaling pathway. Ggpps regulates the balance between protein geranylgeranylation and farnesylation by changing the ratio of GGPP to FPP, which provides a stage-dependent criterion to guide SSC differentiation during the first round of spermatogenesis. Therefore, our study elucidates a potential regulation mode of differentiation and provides a basis for primary male sterility treatment, which is caused by SSC exhaustion and germ cell depletion.

## Materials and Methods

### Mice

Mice with a germ cell–specific and diploid spermatocyte-specific *Ggpps* deletion were generated by crossing Ddx4-Cre and Prm1-Cre transgenic mice to *Ggpps*–LoxP-targeted mice, respectively^49^,^50^. Genotyping was performed using PCR (the primers for PCR and qRT-PCR analyses are listed in Table S1. The knockout efficiency was verified using qRT-PCR to detect Ggpps mRNA and western blotting to detect Ggpps protein in isolated germ cells (Fig. S1). Rapamycin (Selleck Chemicals, USA) and vehicle were intraperitoneally (i.p.) injected every 24 hours from postnatal days 3 to 13. The concentration of rapamycin was 1 mg/ml in 0.5% sodium carboxymethyl cellulose (CMC), which injected at a dose of 4 mg/kg/day. And vehicle was 0.5% CMC in water, which given at a dose of the same volume as rapamycin.

All animal procedures were performed in accordance with the Animal Care and Use Committee of the Model Animal Research Center of Nanjing University. Animal welfare and experimental procedures were carried out in accordance with the Guide for the Care and Use of Laboratory Animals (Ministry of Science and Technology of China, 2006) and related ethical regulations of the Model Animal Research Center of Nanjing University. All experimental protocol were approved by Model Animal Research Center of Nanjing University, including any relevant details.

### Fertility assay and epididymal sperm counting

For reproductive capacity tests, six unique 8-week-old C57BL/6 females were housed with control or Ggpps mutant males. Female mice were checked for vaginal plugs to confirm successful mating. Litter sizes were recorded for statistical analyses. For sperm counting, epididymides were removed and minced in M2 medium (Sigma-Aldrich, St. Louis, MO) containing 3% BSA and incubated at 37 °C for 30 min to release sperm into the medium. The total sperm count in the suspension using a hemacytometer under light microscopy^51^.

### Histology, immunofluorescence and TUNEL assays

For hematoxylin & eosin (H&E) staining, testes were fixed in Bouin’s fixative and processed for gradient dehydration and paraffin-embedding for sectioning. The Sertoli cells, spermatogonia and spermatocytes were identified according to their morphological characters and their location within the seminiferous tubule^29^. The numbers of different cell types in each tubule section were counted, normalized to sertoli cell and presented as the mean ± s.e.m from five groups of control and knockout mice. For immunofluorescence, testes were fixed in 4% PFA and processed for gradient dehydration and paraffin-embedding for sectioning. The primary antibodies for immunofluorescence analyses were as follows: rabbit anti-MVH/DDX4 (1:100; Abcam, Cambridge, MA), rabbit anti-WT1 (1:200; Santa Cruz, CA), mouse anti-Plzf (1:200; Santa Cruz, CA) and mouse anti-Sycp3 (1:200; Abcam, Cambridge, MA). Alexa Fluor 488 or Alexa Fluor 594 (Invitrogen, Carlsbad, CA) was used as a secondary antibody. Apoptosis was assessed using a fluorescent TUNEL kit (Promega, Madison, WI). All images were visualized and captured with a light microscope (Olympus BX51, Olympus, Melville, NY) connected to a DP71 camera (Olympus Corp., Tokyo, Japan) that was controlled by Image-Pro software. The number of positively marked cells in each tubule section were counted, normalized to sertoli cell and presented as the mean ± s.e.m of from five groups of control and knockout mice.

### Isolation, enrichment and culture of Thy1^+^ germ cells

Testes from control and knockout mice were collected at specific time and were digested in Trypsin-EDTA and DNase I to produce a single-cell suspension. Sertoli cells and Leydig cells were eliminated by centrifugation through a 30% Percoll solution to obtain a purified spermatogenetic cell suspension. The coarse purified spermatogenetic cell suspension was incubated with the Thy-1 antibody conjugated to magnetic microbeads (Miltenyi Biotec, Auburn, CA). Magnetic-activated cell sorting (MACS) separation was performed to obtain testis cells enriched for SSCs^52^. The enriched SSCs were cultured on SIM mouse embryo-derived thioguanine- and ouabain-resistant (STO) feeder layer cells in serum-free medium (SFM) supplemented with penicillin (100 U/ml) and streptomycin (100 mg/ml). Recombinant human GDNF, rat GFRa1, and human bFGF were added to a final concentration of 20 ng/ml, 150 ng/ml, and 1 ng/ml, respectively. The components of SFM was according to Hiroshi Kubota and Ralph L. Brinster^52^ and attached in our supplemental materials (Tables S2 and S3). All-trans RA (Sigma-Aldrich, St. Louis, MO) was used at a final concentration of 1 μM to induce spermatogonia differentiation. GGPP, FPP (Sigma-Aldrich, St. Louis, MO) were each used at a final concentration of 10 μM. Cells were maintained in a humidified atmosphere containing 5% CO_2_ at 37 °C for 48 h.

### Immunoprecipitation and immunoblotting

Purified spermatogenic cells were harvested in RIPA buffer containing vanadate and protease inhibitors. The lysates (250 mg) were immunoprecipitated with anti–Rheb antibodies (Santa Cruz, CA) at 4 °C overnight before adding agarose bead-coupled protein A (GE Healthcare). For immunoblotting, cell and tissue protein extracts (250 mg total protein) were boiled in loading buffer, separated by 10–12% SDS-PAGE, and transferred onto polyvinylidene difluoride membranes (Roche Diagnostics). The membranes were incubated overnight with the appropriate primary antibodies. The bound antibodies were visualized using alkaline phosphatase-conjugated secondary antibodies. Band intensities were quantified using ImageJ.

### Membrane association measurements, farnesylation and Rheb activation assay

Spermatogenic cell pellets were lysed, homogenized, and ultracentrifuged to fractionate membrane proteins and cytoplasmic proteins^53^. These subcellular fractions were quantified and adjusted to 400 ng and immunoprecipitated using the Rheb antibody, which were subsequently subjected to western blot analysis to determine the proteins that were present in each fraction. Rheb activation was assessed using a Rheb Activation Assay Kit (New East Biosciences).

### RNA extraction and gene expression

RNA was extracted from mouse testes, purified testicular cells, and primary germ cells for qRT-PCR analyses. SYBR Green Master Mix (Takara Bio Inc.) was used in an ABI 7300 system (Applied Biosystems, Carlsbad, CA). Primer sequences are listed in Supplementary Table 1.

### HPLC-MS/MS measurement of FPP and GGPP

Spermatogenetic cells were isolated and enriched using MACS with a Thy-1 antibody from 6 d and 8 d CTL and *Ggpps*^*−/−*^ Ddx4-Cre mouse testes, snap-frozen in liquid nitrogen and stored at −70 °C. FPP and GGPP levels were detected as described previously^13^ using an LCMS-8040 (Shimadzu Corporation, Kyoto, Japan) in the negative electrospray ionization mode.

### Data analysis

All data were presented as the mean ± s.e.m. Statistical comparisons were performed with an unpaired two-tailed Student’s t-test. In all cases, statistical significance was indicated as P < 0.05 (one asterisk) or P < 0.01 (two asterisks).

## Additional Information

**How to cite this article**: Diao, F. *et al*. Alteration of protein prenylation promotes spermatogonial differentiation and exhausts spermatogonial stem cells in newborn mice. *Sci. Rep.* **6**, 28917; doi: 10.1038/srep28917 (2016).

## Supplementary Material

Supplementary Information
