# Supplementary material for: Alteration of protein prenylation promotes spermatogonial differentiation and exhausts spermatogonial stem cells in newborn mice
Source: Sci Rep. 2016 Jul 4;6:28917. doi: 10.1038/srep28917 (PMC4931501; doi:10.1038/srep28917)
Supplement: Supplementary Information [file srep28917-s1.pdf]

**Supplementary section to:**

# Alteration of protein prenylation promotes spermatogonial differentiation and exhausts spermatogonial stem cells in newborn mice

**Fan Diao[1, #], Chen Jiang[1, #], Xiu-Xing Wang[1], Rui-Lou Zhu[1], Qiang Wang[1], Bing Yao[2*], Chao-Jun Li[1*]**

[1]MOE Key Laboratory of Model Animals for Disease Study, Model Animal Research Center and the Medical School of Nanjing University, National Resource Center for Mutant Mice, Nanjing 210061, China; [2]Center of Reproductive Medicine, Nanjing Jinling Hospital, the Medical School of Nanjing University, Nanjing 210002, China.

[#]These authors contributed equally to this work.

*Corresponding and requests for materials should be addressed to: B.Y. (yaobing@nju.edu.cn) or CJ L. (licj@nju.edu.cn)

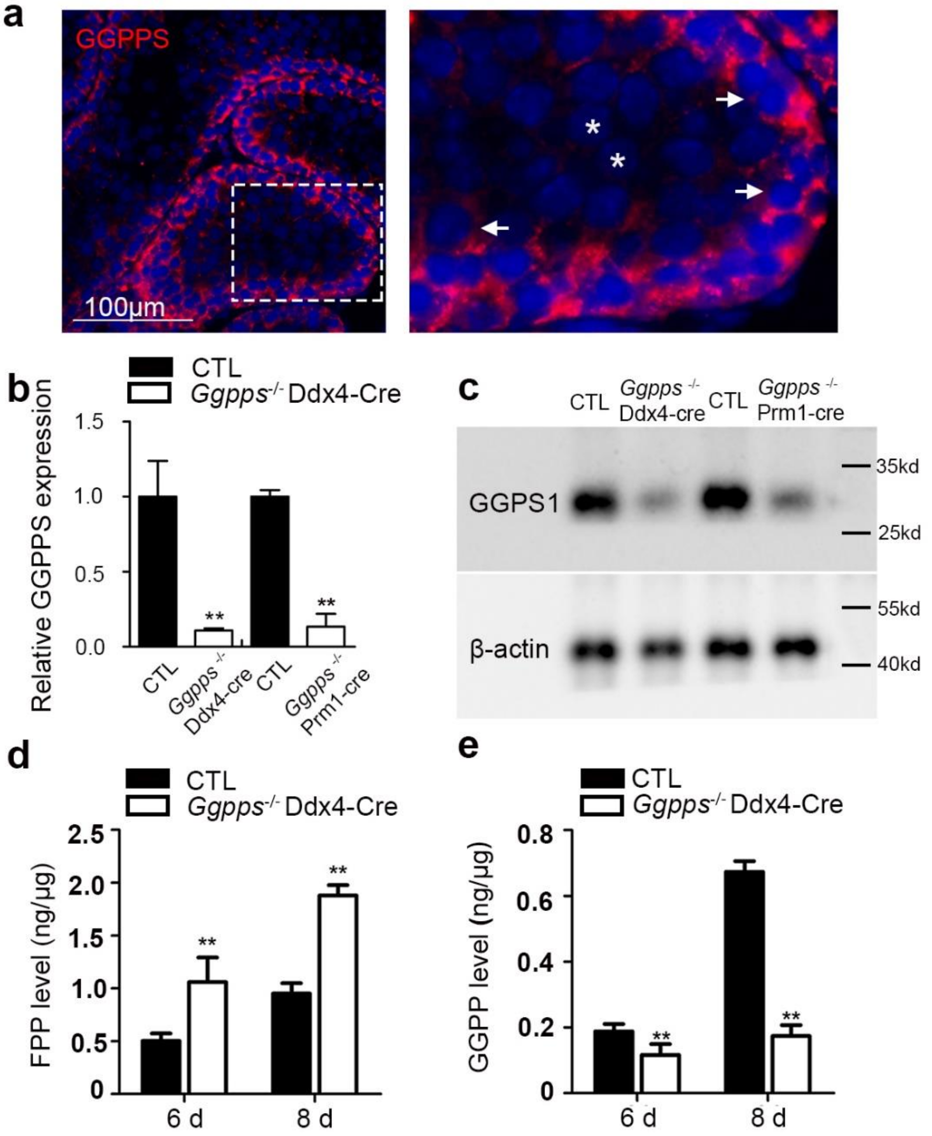


**Supplementary Figure 1**.

(a) Ggpps  expression pattern of wild-type mouse testes at postnatal day 24 shows by immunofluorescence. Spermatogonia and primary spermatocytes labeled by arrows and secondary spermatocytes labeled by asterisks. Scale bar=100 μm. (b,c) Relative mRNA expression and western blotting for protein of Ggpps in isolated germ cells of control and knockout mice. (d, e) HPLC–MS/MS analysis of GGPP and FPP levels at postnatal 6 d and 8 d, showing GGPP deficiency and FPP accumulation in Thy1$^{+}$ spermatogenetic cells of Ggpps$^{-/-}$Ddx4-Cre mice. FPP 6d p**=8.21E-06, 8d p**=5.25E-06, GGPP 6d p**=3.32E-06, 8d p**=7.33E-09.

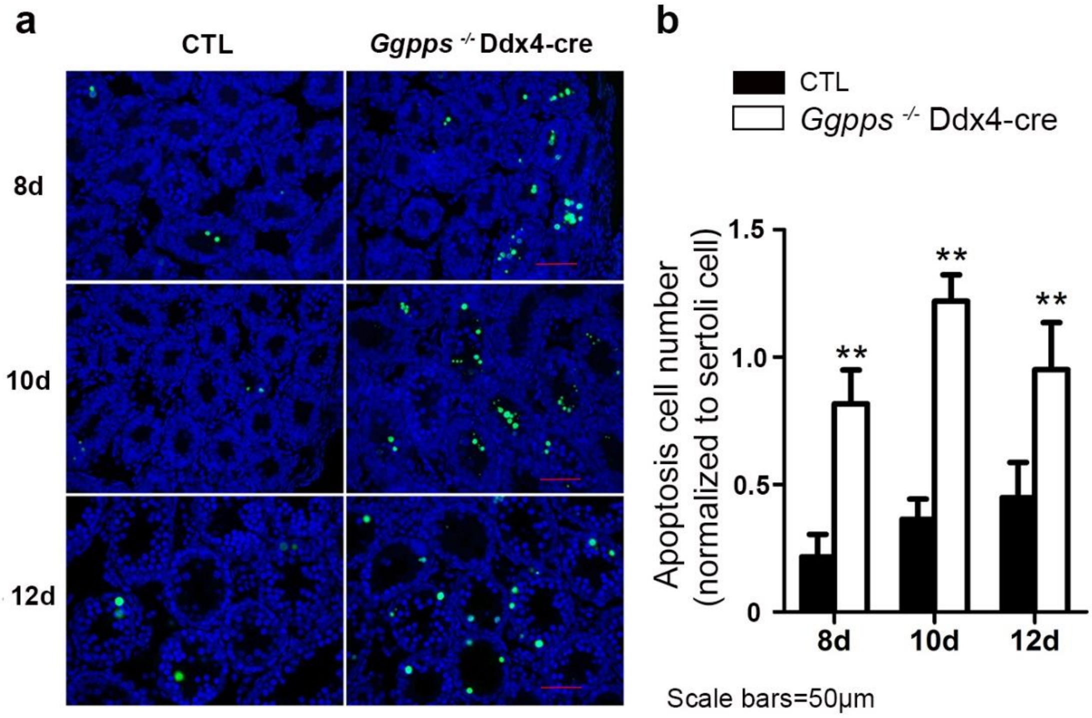


**Supplementary Figure 2. Apoptosis analysis of CTL and *Ggpps*$^{-/-}$ Ddx4-Cre mice.**

(a) TUNEL staining of seminiferous tubules from 8d to 12d. Scale bars =50 μm

(b) Statistical analysis of apoptotic cell number per 20 tubules section. n=6,8d p**=3.57E-04, 10d p**=2.81E-04, 12d p**=5.93E-04.

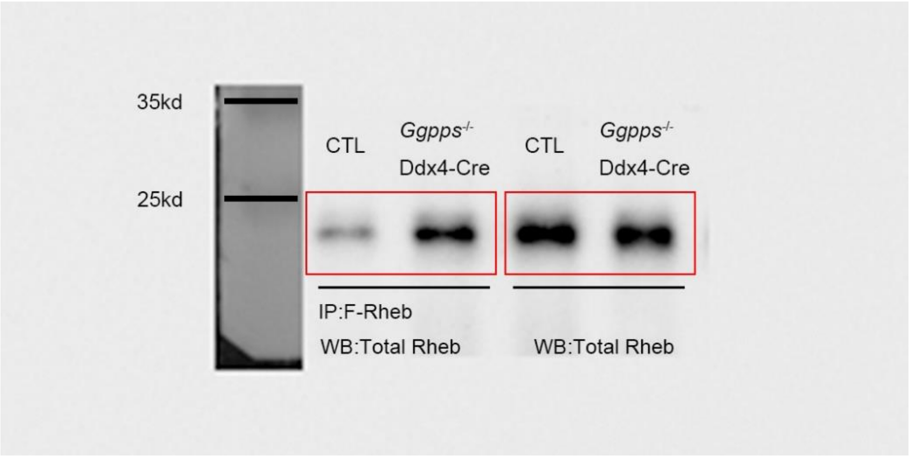


**Supplementary Figure 3. Full-length images of the immunoblots in Figure 5a.**

Red line boxes indicate the cropped images used in Figure 5a.

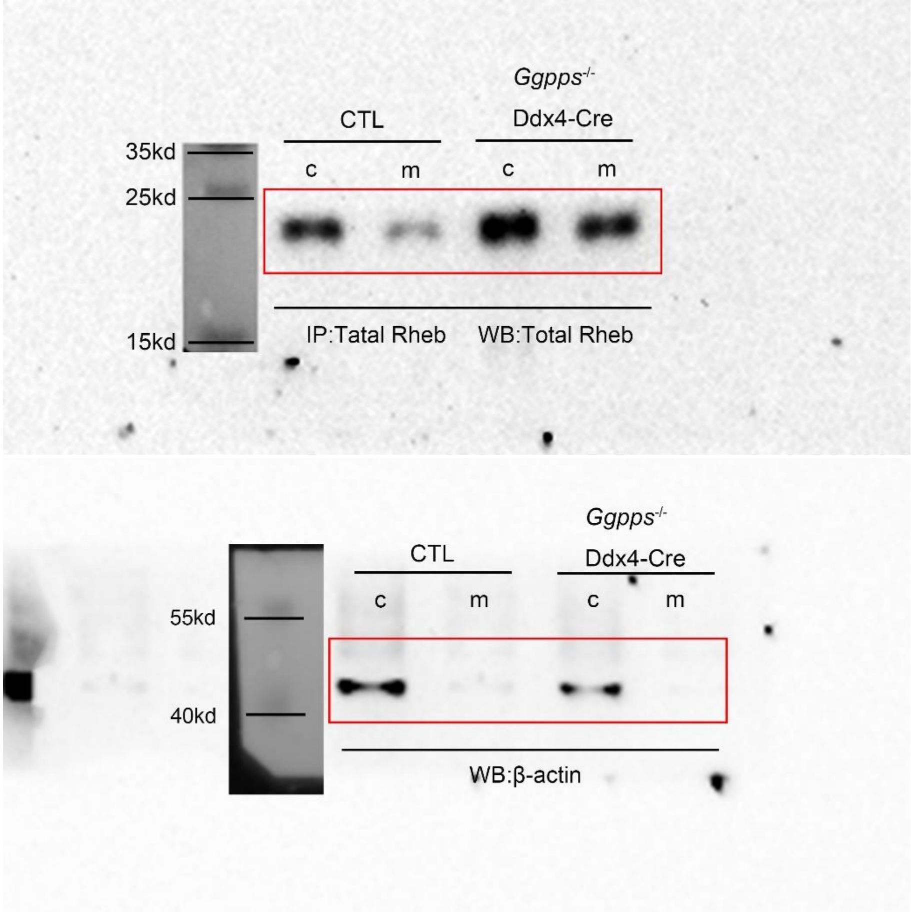


**Supplementary Figure4. Full-length images of the immunoblots in Figure 5b.**
Red line boxes indicate the cropped images used in Figure 5b. β-actin was used as an internal control.

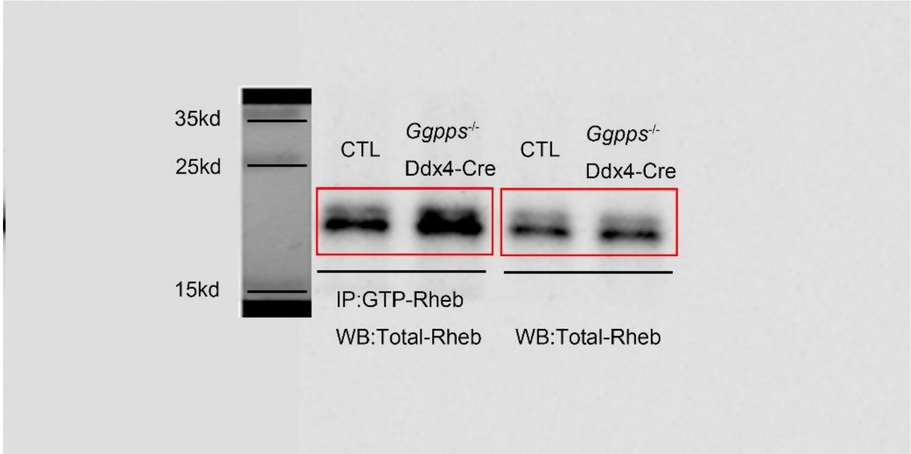


**Supplementary Figure 5. Full-length images of the immunoblots in Figure 5c.**
Red line boxes indicate the cropped images used in Figure 5c.

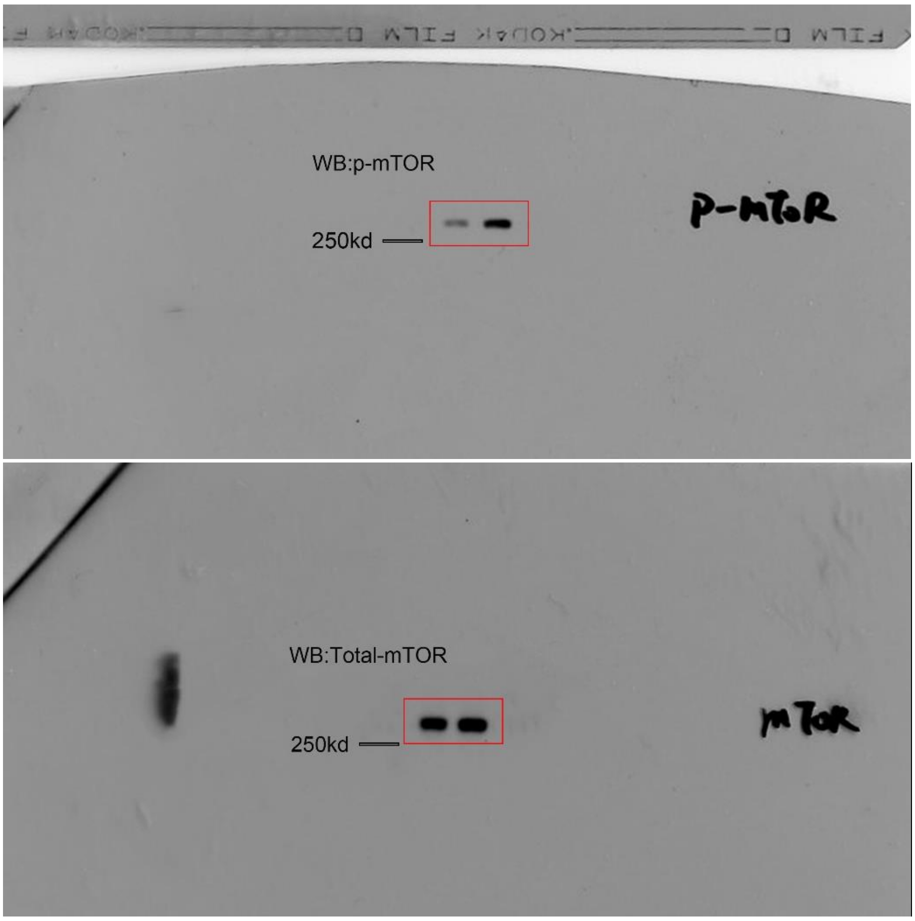


**Supplementary Figure 6. Full-length images of the immunoblots in Figure 5d.**
Red line boxes indicate the cropped images used in Figure 5c.

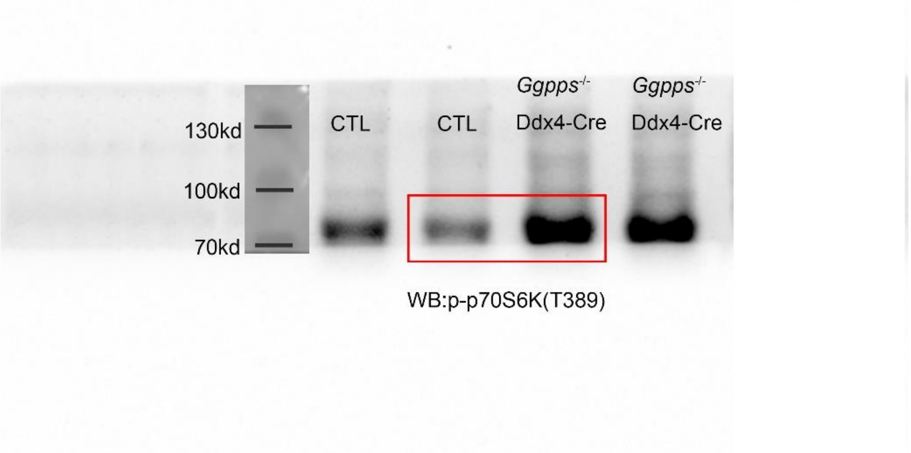

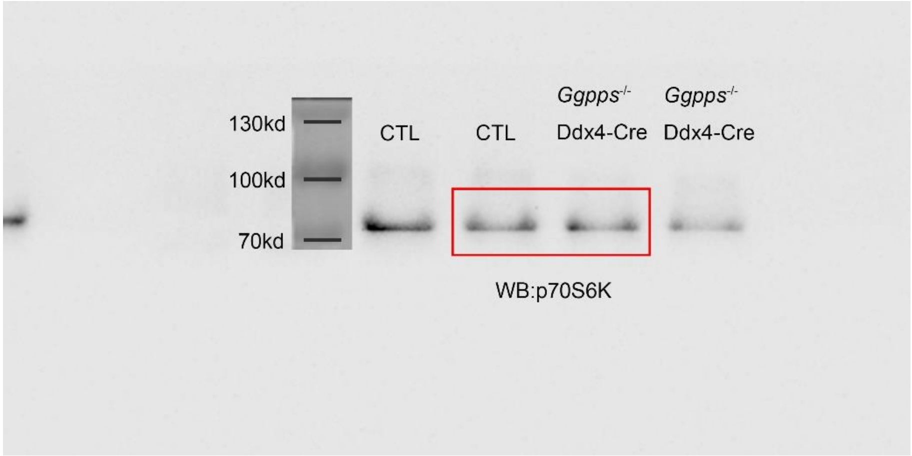


**Supplementary Figure 7. Full-length images of the immunoblots in Figure 5e.**
Red line boxes indicate the cropped images used in Figure 5e.

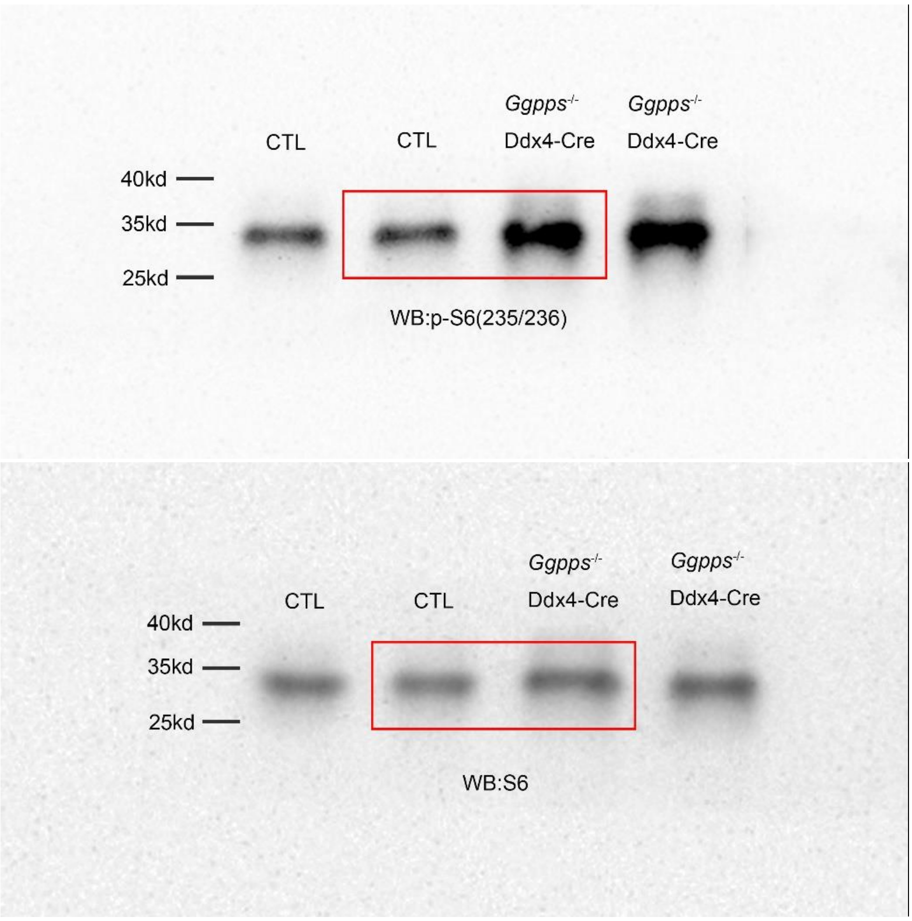


**Supplementary Figure 8. Full-length images of the immunoblots in Figure 5f.**
Red line boxes indicate the cropped images used in Figure 5f.

| Gene symbol | Forward primer | Reverse primer |
|---|---|---|
| Cre | 5’-TGC CACGACCAAGTGACAGCAATG-3' | 5’-AGAGACGGAAATCCATCGCTCG-3' |
| Loxp | 5’-AATTGTGTGTGGTAGGGGTA-3' | 5'-AACTTGCTTCAGAACTGAGC-3' |
| GGPPS | 5’-TTCACCAACACCTGTAACTC-3' | 5'-TTATTGACAAGCCCAGAGC-3' |
| c-kit | 5'-GCCTGACGTGCATTGATCC-3' | 5'-AGTGGCCTCGGCTTTTTCC-3' |
| Stra8 | 5'-ACCCTGGTAGGGCTCTTCAA-3' | 5'-GACCTCCTCTAAGCTGTTGGG-3' |
| SYCP3 | 5'-AGCCAGTAACCAGAAAATTGAGC-3 | 5'-CCACTGCTGCAACACATTCATA-3 |

**Supplementary Table 1. Primer sequences used for PCR and qRT-PCR**

| | Manufacturer | Catalog number | Concentration |
|---|---|---|---|
| MEM a | Invitrogen | 12561 | Basal media |
| Penicillin | Gibco/Invitrogen | 15140 | 50 units/ml |
| Streptomycin | Gibco/Invitrogen | 15140 | 50 m g/ml |
| BSA | Sigma | A3803 | 0.20% |
| Transferrin | Sigma | T 1283 | 10 mg/ml |
| FFA mixture | Sigma | See Table 3 | 7.6 meq/L |
| $Na_2SeO_3$ | Aldrich/Sigma | 481815 | $3\times10^{-8}$ M |
| L -glutamine | Gibco/Invitrogen | 25030 | 2 mM |
| 2-ME | Sigma | M 7522 | 50 μM |
| Insulin | Sigma | I 5500 | 5 μg/ml |
| HEPES | Sigma | H 0887 | 10 mM |
| Putrescine | Sigma | P 5780 | 60 mM |

**Supplementary Table 2. Components in Mouse Serum-Free Medium (SFM)**

| Free fatty acid (FFA) | Manufacturer | Catalog number | Stock solution | 100 meq/l FFA mixtureb |
|---|---|---|---|---|
| Linolenic acid | Sigma | L 2376 | 1M | 5.6 m l (5.6 mM) |
| Oleic acid | Sigma | O 1008 | 1M | 13.4 m l (13.4 mM) |
| Palmitoleic acid | Sigma | P 9417 | 1M | 2.8 m l (2.8 mM) |
| Linoleic acid | Sigma | L 1012 | 1M | 35.6 m l (35.6 mM) |
| Palmitic acid | Sigma | P 0500 | 1M | 31.0 m l (31.0 mM) |
| Stearic acid | Sigma | S 4751 | 151 mM | 76.9 m l (11.6 mM) |
| Absolute ethanol | | | | 834.7 m l |
| Final volume | | | | 1000 m l (100 meq/l) |

**Supplementary Table 3. Preparation of Free Fatty Acid Mixture**
